# Cerebrospinal fluid rhinorrhea in a bilateral frontal decompressive craniectomy patient caused by strenuous activity: A case report

**DOI:** 10.1097/MD.0000000000013189

**Published:** 2018-11-21

**Authors:** Guangming Wang, Lichao Sun, Wenchen Li, Jinlu Yu

**Affiliations:** a Department of Neurosurgery; b Department of Emergency, First Hospital of Jilin University, Changchun, PR China.

**Keywords:** cerebrospinal fluid rhinorrhea, iatrogenic cranial defect

## Abstract

**Rationale:**

Iatrogenic cerebrospinal fluid (CSF) rhinorrhea in a bilateral frontal decompressive craniectomy patient triggered by strenuous sport is rare. To the best of our knowledge, no similar case has yet been reported.

**Patient concerns:**

Herein, we report a case of CSF rhinorrhea in a 37-year-old man. He had previously suffered a traumatic brain injury in a traffic accident, and a subsequent bilateral frontal decompressive craniectomy operation was performed. Based on the frontal skull defect peculiarity, strenuous exercise may have caused drastic CSF pressure waves to tear the dura mater of the anterior skull base, resulting in CSF rhinorrhea.

**Diagnoses:**

The thin-slice computerized tomography (CT) images revealed a frontal skull defect and the open frontal sinus. In addition, in the opened frontal sinus, low-density liquid-filled areas were visible.

**Interventions:**

During surgery, the torn dura was carefully repaired, and the frontal sinus was filled with temporal muscle, fascia, and fibrin glue. A simultaneous cranioplasty was performed.

**Outcomes:**

The patient was followed-up postoperatively for 12 months to date without rhinorrhea recurrence. Recovery was uneventful.

**Lessons:**

Patients with skull defects should avoid strenuous sports, and cranioplasty should be performed as early as possible in order to decrease the likelihood of a dural tear and prevent the occurrence of CSF leakage. After cranioplasty, the skull should be restored to a closed state to reduce the damaging effects of CSF waves during movement. It is important to maintain normal intracranial pressure to reduce the recurrence rate of CSF rhinorrhea.

## 1 Introduction

Cerebrospinal fluid (CSF) rhinorrhea is a common brain condition that is treated with neurosurgery. Among nontraumatic CSF fistulas, spontaneous fistulas are the most commonly reported at 41% of all fistulas, other types of CSF fistulas are related to iatrogenic trauma after surgery (30%), tumors (5%), or congenital malformation of the skull base (3%).^[1]^ Regardless of the type, CSF rhinorrhea occurs when there is an osseous and dural defect at the skull base and direct communication with the extracranial space, usually a paranasal sinus.^[2]^

Ommaya et al reported that all cases of CSF rhinorrhea, even spontaneous CSF rhinorrhea, had an underlying cause.^[3]^ We report the case of a patient who developed bacterial meningitis caused by the leakage of CSF into the frontal sinus. The patient had a bilateral iatrogenic frontal skull defect as the basis of the pathological anatomy, and strenuous exercise was the initiating factor that led to a tear in the dura.

## 2 Case presentation

A 37-year-old man who was transferred to our hospital presented with a one-week history of CSF rhinorrhea, a four-day history of fever and a one-day history of headache. Six months prior to admission, he had suffered a traumatic brain injury in a traffic accident. Brain computed tomography (CT) revealed bilateral frontal lobe contusions and multiple fractures of the bilateral frontal bones. Under general anesthesia, an emergency contusion cleaning procedure and bilateral frontal decompressive craniectomy were performed.

**Figure 1:**
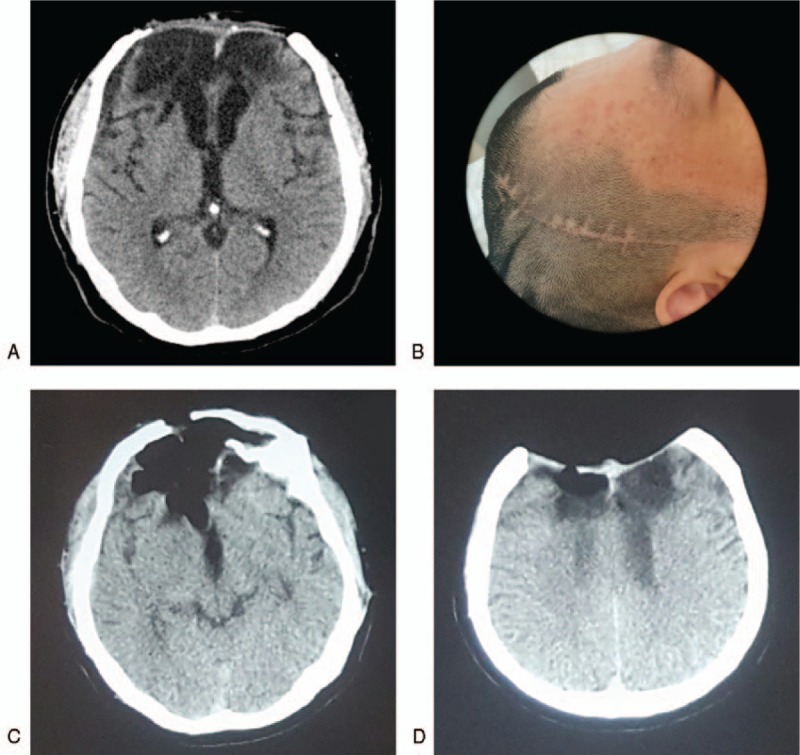
Images of the patient at admission. A: Head CT showed bilateral frontal bone defects and lobe necrosis; B: The surgical scar and a significantly sunken scalp were noted; C–D: Head CT showed intracranial pneumocephalus. CT = computerized tomography.

Postoperatively, the patient had a favorable recovery. Head CT showed bilateral frontal bone defects and brain necrosis, and a significantly sunken scalp was noted (Fig. 1A and B). However, one week prior to the present admission to our hospital, he developed CSF rhinorrhea 20 min after jumping rope, along with a subsequent 4-day fever. At admission, a brain CT revealed an intracranial pneumatocele (Fig. 1C and D).

At admission, he was drowsy upon physical examination. His temperature was 37.6°C. Neck stiffness and meningeal irritation were observed. A laboratory examination revealed an elevated C-reactive protein level of 126 mg/L and a peripheral leukocytosis of 20.1 × 10^9^/L. A CSF examination revealed pleocytosis (287 × 10^6^ cells/L, of which 80% were polymorphonuclear cells), increased total protein (998 mg/L) and a Pandy test result of ++. *Streptococcal pneumonia* was detected in a bacterial culture of the CSF sample.

**Figure 2:**
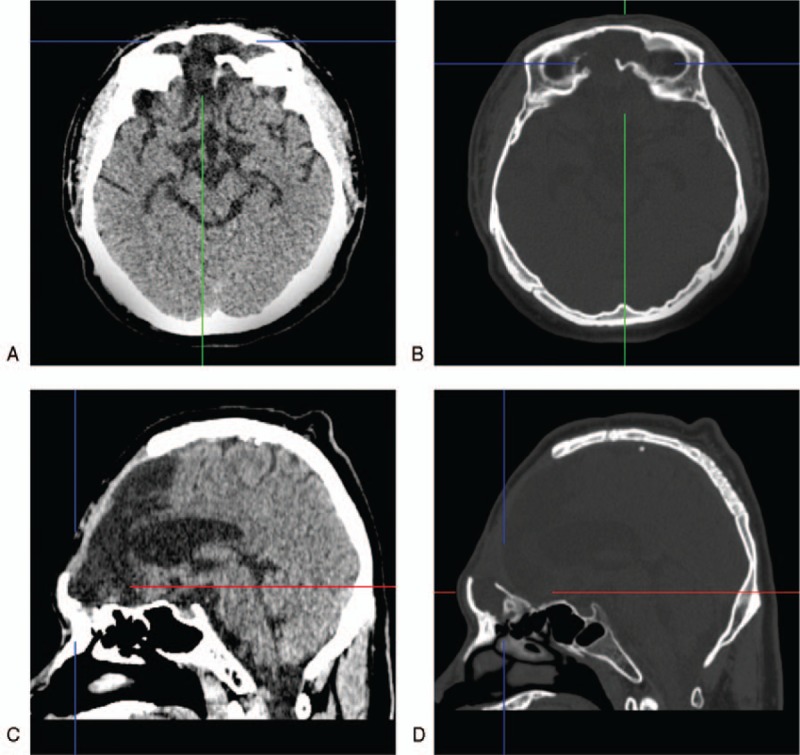
Thin-slice CT images. A–D: The axial and sagittal CT revealed the open frontal sinus. Low-density liquid-filled areas are visible in the opened frontal sinus. CT = computerized tomography.

The patient was therefore treated with high-dose ceftriaxone. Two weeks later, the patient's symptoms returned to normal. Three CSF examinations showed normal results. Two months after the onset of CSF rhinorrhea, the patient still had persistent unilateral clear nasal drainage that worsened when standing or sitting. Axial and sagittal CT revealed an open frontal sinus. In addition, low-density areas indicating liquid were visible in the opened frontal sinus (Fig. 2).

A cranioplasty and dural defect repair were planned. A bicoronal scalp incision was made along the primary surgical incision, and a scalp flap was carefully elevated. The frontal sinus was filled with CSF, and the dural tear was identified. After opening the dura, a hyperplastic gliosis scar was visible around the area of the original contusion. We excised the glial scar. Wide apposition of the whole region of the anterior skull base including the ethmoidal roof, orbital roof, and lateral walls extending to the orbital apex was performed.

**Figure 3:**
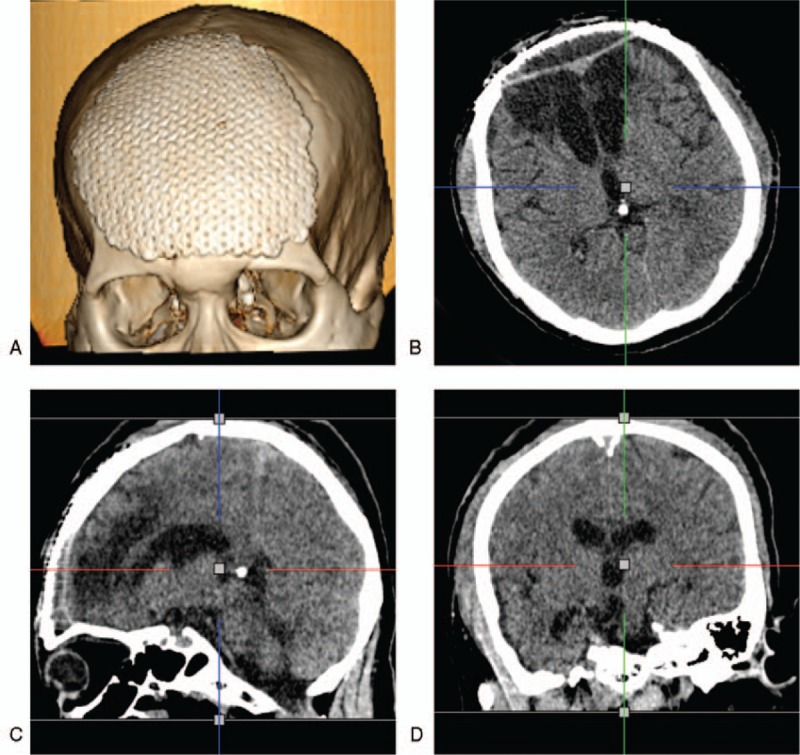
CT images after cranioplasty. A–D: 3D reconstruction and thin-slice CT showed the appropriate positioning of the metal titanium plate and normal brain ventricles. CT = computerized tomography.

After careful examination of the anterior skull base, no other leaks were found. The dural defect was repaired with artificial dural mater. The frontal sinus was filled with temporal muscle, temporalis fascia, and fibrin glue. Simultaneous cranioplasty was performed using a customized titanium mesh (Fig. 3). The operation was uneventful. During a follow-up period of 12 months, the patient's recovery was satisfactory.

## 3 Ethics statement

As a case report with written consent, our institution does not require formal ethical approval. Written informed consent was obtained from the patient and his wife for publication of this case report.

## 4 Discussion

CSF rhinorrhea after craniofacial trauma mainly occurs in the nasal cavity, and CSF can enter the nasal cavity through defects in both the dura mater and bone in relation to the frontal ethmoid or sphenoid sinuses or along the cribriform plate for multiple reasons.^[4]^ Ommaya et al postulated that there are areas of focal atrophy in the cribriform plate and sella that become filled with a pouch of CSF. Normal CSF pressure waves cause these pouches to enlarge, erode and eventually rupture out of the bone.^[3]^

In our case, the patient had an iatrogenic frontal defect, and the roof of the frontal sinus was open and filled with a large pouch of CSF (Fig. 2). This was the pathological basis for the CSF leakage, and the brain tissue in this region was not protected by the skull. Based on this anatomical characteristic, when the patient performed strenuous exercises, such as jumping rope or running, the soft tissue of the anterior cranial fossa was relatively displaced, leading to a traumatic CSF wave pounding the skull base.

**Figure 4:**
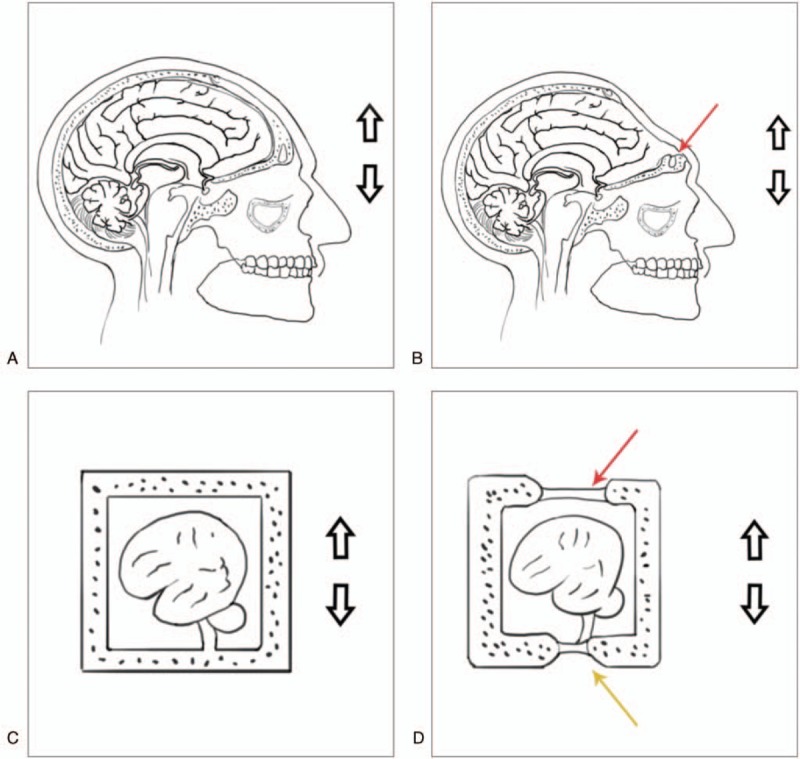
The mechanism of CSF rhinorrhea. A: Normal closed skull; B: Patient with an iatrogenic frontal defect. The red arrow shows the opened frontal sinus and the dura mater covering the defect in the frontal sinus. The red arrow shows the frontal bone defect; C and D: Illustrations of the frontal shift phenomenon in strenuous exercise. The brain tissue in the anterior cranial fossa may be relatively displaced in strenuous exercise, causing a considerable pressure wave at the skull base. The red arrow shows the defect in the skull roof covered by the scalp and dura mater. The yellow arrow shows the skull base covered by the dura mater. CSF = cerebrospinal fluid.

The CSF pressure wave was much larger than that caused by normal brain pulsation, and it ultimately lacerated the dura mater at the skull base. Finally, the dura mater overlying the open frontal sinus was torn, and CSF leaked into the frontal sinus and nasal cavities. Therefore, in patients who have skull defects, strenuous sports should be avoided, and cranioplasty should be performed as early as possible to decrease the probability of dural tears and prevent the occurrence of CSF leakage. The mechanism is shown in Figure 4.

The use of CT for evaluating skull base defects has become a mainstay in the work-up of CSF fistulas due to its ability to resolve osseous structures.^[2,5]^ In particular, the development of CT thin-section and 3D reconstruction technology enables the identification of smaller cranial defects.^[2]^ In our case, the CT scan allowed clear visualization of the location of the CSF rhinorrhea. The patient was misdiagnosed with a cold, upper respiratory infection or rhinitis due to his major complaints of intermittent serous nasal discharge, fever and headache.^[6,7]^

Communication with the intracranial space increases the risk of pneumocephalus, meningitis and brain abscessation, which are potentially lethal.^[8]^ The mortality rate of meningitis caused by CSF rhinorrhea is 14%.^[9]^ The organisms that lead to ascending meningitis or meningoencephalitis typically include flora from the upper respiratory tract. *Streptococcus pneumoniae* is the most common, and this organism has been implicated in recurrent bacterial meningitis in this patient population.^[10]^

Because of the attendant risk of meningitis, brain abscessation, and pneumocephalus, all patients with persistent CSF rhinorrhea should undergo surgery if conservative treatment has failed.^[11]^ Patients with persistent CSF rhinorrhea require a surgical approach.^[12]^ Endoscopic endonasal fistula repair is safe and has a high success rate.^[13]^ Intracranial access (craniotomy) is utilized where there are skull fractures requiring reconstruction, extensive skull base fractures, intracranial hemorrhages and contusions requiring treatment or sizeable deficits of the dura, for which the probability of relapse is high.^[14,15]^

Iatrogenic CSF leakage always occurs following surgery of the skull base; however, in our patient, CSF rhinorrhea was fundamentally caused by the opening of the frontal sinus during forehead surgery and was triggered by strenuous sport, which is different from other skull defects leading to iatrogenic CSF rhinorrhea. This result suggests to us that sports should be avoided for this population of patients. This case should also remind neurosurgeons that the frontal sinus should be adequately repaired and filled during bilateral frontal decompressive craniectomy.

Given the characteristics of the large frontal bone defect in our patient, the craniotomy was used to resolve the CSF rhinorrhea, and simultaneous cranioplasty was performed. During the rhinorrhea repair surgery and because of the defects in the skull and the clearance of the frontal lobe, we could better expose the anterior cranial fossa including the frontal sinus and sieve plate. This allowed direct visualization of the defect for a satisfactory closure.^[16]^

The source of CSF leakage is often repaired with autologous transplantation material, such as cartilage, inner thigh muscle or bone, nasal septum mucosa, turbinate bone, fascia, abdominal fat, ear cartilage, and other autologous tissues.^[4,8,17]^ During surgery, we used temporalis muscle and fascia to avoid trauma caused by a separate incision.

Recurrence of CSF rhinorrhea is not uncommon. In our case, over a follow-up period of 12 months, there was no recurrence of CSF rhinorrhea. This lack of recurrence may be related to the adequate filling and repair of the opened frontal sinus. We also assume that the cranioplasty that closed the cranial cavity may play a role. This maintained the patient's intracranial pressure, reducing the damaging effects of the CSF waves generated during movement.

## 5 Conclusion

To the best of our knowledge, no similar case has been reported before. Iatrogenic CSF rhinorrhea should be treated with surgery. Careful and thorough determination of the leakage location and identification of any defects are the keys to a successful surgery. For cranial defect, especially in the frontal region, strenuous exercise may cause strong CSF pressure waves to tear the dura mater and thus should be avoided. Cranioplasty should be performed as early as possible to decrease the probability of a dural tear and to prevent CSF leakage. After cranioplasty, it is important to maintain normal intracranial pressure to reduce the recurrence rate of CSF rhinorrhea.

## Author contributions

Development of the idea and performance of the surgical operation: Guangming Wang.

Development of the idea and editing of the final draft: Jinlu Yu.

Data collection and drafting of the original article: Lichao Sun.

Drafting of the original article: Wenchen Li.

**Data curation:** Lichao Sun, Wenchen Li.

**Project administration:** Guangming Wang, Jinlu Yu.

**Writing – original draft:** Lichao Sun, Wenchen Li.

**Writing – review and editing:** Jinlu Yu.
